# Warfarin‐related nephropathy: A case report of a delayed renal function improvement

**DOI:** 10.1002/ccr3.4105

**Published:** 2021-05-19

**Authors:** Eriko Nonaka, Tsuyoshi Takashima, Keiichirou Matsumoto, Makoto Fukuda, Shuichi Rikitake, Motoaki Miyazono

**Affiliations:** ^1^ Department of Nephrology Faculty of Medicine Saga University Saga Japan; ^2^ Department of Nephrology NHO Ureshino Medical center Saga Japan

**Keywords:** acute kidney injury, anticoagulant‐related nephropathy, warfarin‐related nephropathy

## Abstract

We experienced a case in which improving the renal damage caused by warfarin‐related nephropathy took a long time. It is important to follow up for a long time after the initiation of dialysis due to warfarin‐related nephropathy.

## Graphical abstract

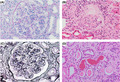

## 1 BACKGROUND

Warfarin‐related nephropathy (WRN) is a type of acute kidney injury (AKI) that may be caused by excessive anticoagulation with warfarin and other anticoagulants. This is a relatively new disease concept, and there is no consensus regarding the prognosis of the renal function. A 73‐year‐old man with chronic atrial fibrillation taking warfarin for anticoagulant therapy developed AKI with a serum creatinine level of 7.67 mg/dL and gross macrohematuria. The warfarin was withdrawn, and hemodialysis was started. A renal biopsy showed red cell casts in the renal tubules. Based on these findings, the patient was diagnosed with WRN with acute kidney injury. The renal function improved after 4 months, and dialysis was withdrawn. We experienced a case in which improving the renal damage caused by WRN took a long time, and long‐term follow‐up is important after the initiation of dialysis.

Warfarin‐related nephropathy (WRN) is an acute renal injury caused by the excessive action of anticoagulants, such as warfarin. It is characterized by rapidly progressive renal dysfunction and gross hematuria. A renal biopsy is not commonly performed because of abnormal coagulation, but it may be performed to rule out other renal disorders if the renal function is poorly improved. The renal biopsy findings are characterized by a large number of red blood cells and red blood cell casts in the renal tubules and Bowman's capsule, and also the obstruction the renal tubules.^1^ Most patients with WRN experience temporary renal dysfunction that subsequently improves, but some patients develop end‐stage renal failure without an improved renal function.^2^

We herein report a case in which the renal function eventually improved as a result of continued maintenance dialysis for more than 4 months for renal dysfunction induced by WRN.

## 2 CASE PRESENTATION

A 73‐year‐old man was admitted for acute kidney injury (AKI) and dark urine. He had chronic atrial fibrillation, valvular heart disease, and hypertension and had been taking warfarin and antihypertensive drugs for 3 years. His last checkup (3 months before admission) had revealed mild microscopic hematuria without proteinuria and mild renal dysfunction of serum creatinine 1.1 mg/dL. He had noticed gross macrohematuria about 2 weeks before admission. He had undergone a work‐up for the gross macrohematuria 5 days before admission, where severe renal dysfunction had been found. Warfarin had been stopped at that time because the prothrombin time‐international normalized ratio (PT‐INR) was elevated to 3.90. He was then referred to our hospital for the diagnosis and treatment of renal dysfunction.

On admission, the patient was 155.6 cm tall and weighed 49.7 kg, with a blood pressure of 176/94 mm Hg and temperature of 36.8°C. A physical examination did not reveal any abnormalities of the lungs and abdomen, but a systolic murmur was heard at the apex. Mild leg edema was detected. The urine volume was 313 mL per day, and the color was reddish brown.

The laboratory findings were as follows: serum creatinine was 7.67 mg/dL, the estimated glomerular filtration rate (eGFR) was 6.0 mL/min/1.73 m^2^, serum potassium was 6.0 mEq/L, and C‐reactive protein (CRP) was 0.69 mg/dL. His PT‐INR decreased to 2.2 as he discontinued warfarin. The patient was negative for antineutrophil cytoplasmic antibody, antiglomerular basement membrane antibody, and antinuclear antibody. The concentrations of serum complement, such as C3 and C4, were within the normal range. The urine protein‐to‐creatinine ratio (U‐P/U‐Cr) was 6.6 g/Cr, and the urine sediment contained more than 100 red cells per high‐power field (HPF). Computed tomography showed swelling of the right kidney with no urinary tract obstruction.

We suspected AKI due to WRN and followed the patient to observe whether or not the renal function improved once warfarin was discontinued. However, the serum creatinine level increased to 9.0 mg/dL, and oliguria persisted on the 6th hospital day, so hemodialysis was started. The renal function and urine volume did not improve, so a renal biopsy was performed for the differential diagnosis on the 28th hospital day.

**FIGURE 1:**
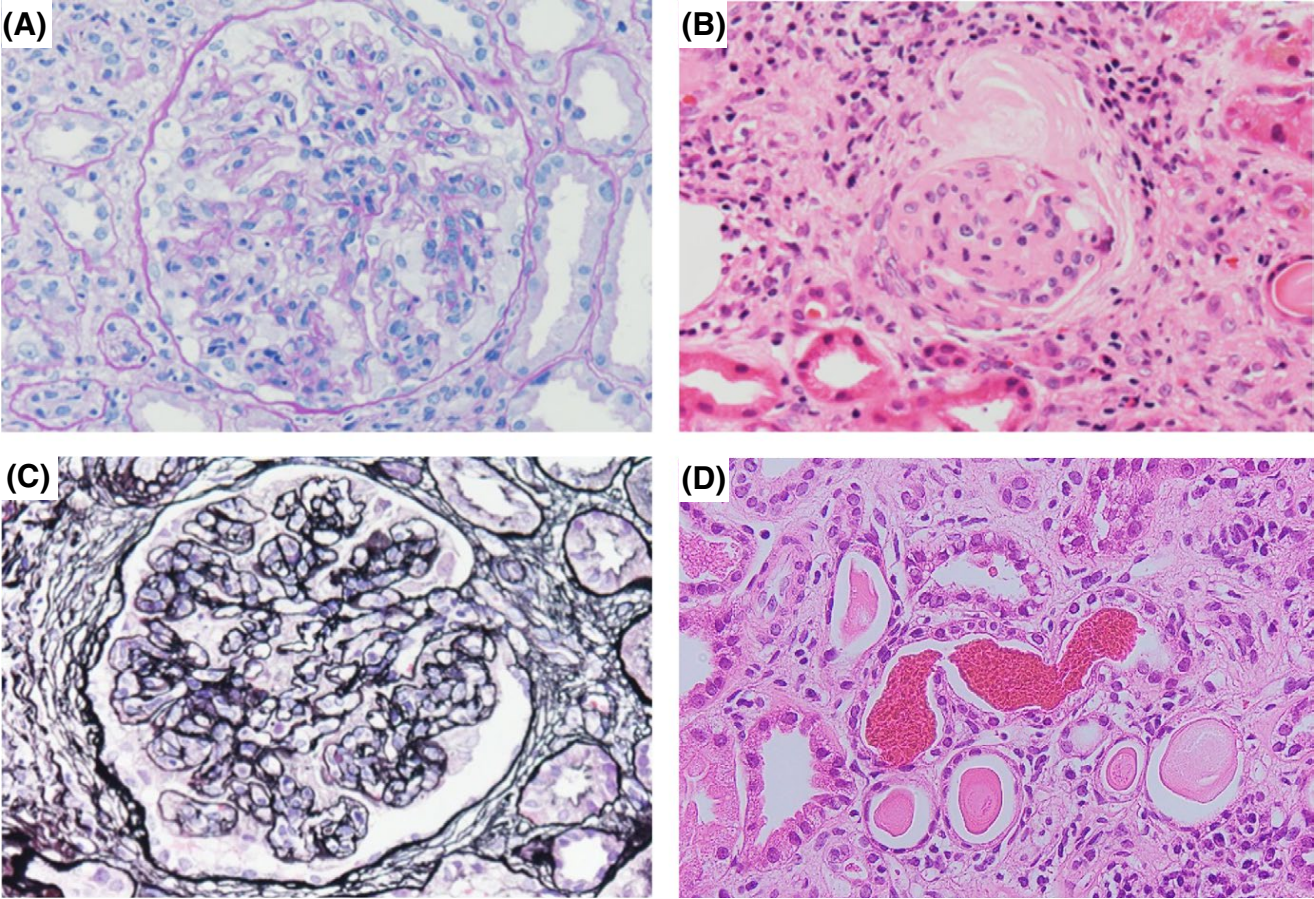
Light microscopic findings of the renal biopsy specimen: A, PAS stain, B, HE stain, C, PAM stain, D, HE stain

A histological analysis revealed that 6.3% (1/16) of the glomeruli showed global sclerosis, and 12.5% (2/16) of the glomeruli showed proliferation of mesangial cells (Figure 1A,B). Thickening of the basement membrane was observed in one glomerulus (Figure 1C). Red blood cell casts and hemorrhaging were observed in 24 renal tubules (Figure 1D). IgA and C3 showed slight deposition. Electron microscopy showed subepithelial edema and no electron‐dense deposits. Based on these histological findings, the patient was diagnosed with AKI with WRN. Mesangial proliferative nephritis may have been present as an underlying disease.

Warfarin‐related nephropathy usually induces AKI occurring during episodes of over‐anticoagulation. However, the renal function did not improve even after discontinuing warfarin, so shunt surgery was performed, and hemodialysis was continued. He was discharged on the 56th hospital day and continued hemodialysis at a clinic near his home. He visited our outpatient hospital to check for changes in the renal function.

**FIGURE 2:**
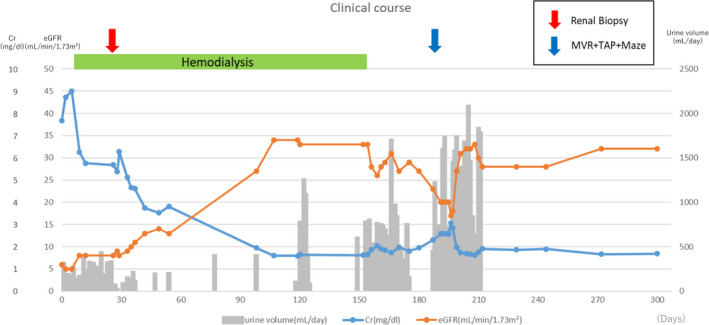
Clinical course. MVR, mitral valve replacement; TAP, tricuspid annuloplasty; Maze, Maze operation

At 2 months after the onset, the urine volume was about 400 mL, and dialysis was continued. At 4 months after the onset, the urine volume was about 1000 mL, and the serum creatinine level was 1.65 mg/dL (a blood test was performed the day after dialysis), so he was hospitalized again to try to stop hemodialysis. At 157 days after the onset, he was withdrawn from dialysis with a serum creatinine level of 1.8‐2.0 mg/dL and good weight control. He started taking edoxaban for chronic atrial fibrillation and had no recurrence of AKI and gross hematuria. At 187 days after the onset, he underwent surgery (mitral valve replacement, tricuspid annuloplasty, and maze operation) for heart disease. After the operation, his renal function declined temporarily but improved thereafter. At 1 year after the onset, the renal function had improved, with a serum creatinine level of approximately 1.6 mg/dL (Figure 2).

## 3 DISCUSSION

Warfarin‐related nephropathy is a type of AKI that may be caused by excessive anticoagulation with warfarin and other anticoagulants. It also referred to as anticoagulant‐related nephropathy (ARN). Wheeler et al reported that disruption of the glomerular filtration barrier leads to hemorrhaging into Bowman's space and the renal tubules.^1^ Erythrocyte casts form in the renal tubules, causing obstruction and ischemia. Vitamin K antagonists other than warfarin also reportedly cause WRN.^3^ Thrombin, the vitamin K‐dependent coagulation factor, binds and activates proteinase‐activated receptors (PARs) expressed in numerous cells, including endothelial cells. It is hypothesized that a reduction in thrombin levels caused by anticoagulants causes the breakdown of the endothelial barrier, allowing glomerular hemorrhaging.^1^

The risk factors for warfarin‐related nephropathy include age, diabetes, heart disease, hypertension, and chronic kidney disease, and the occurrence of WRN was reported to be associated with mortality.^4^, ^5^ The frequency of ARN ranged from 19.3% to 63.0% in previous cohort studies.^6^ There was also a report that the incidence of acute AKI was higher in patients taking warfarin than in those receiving a novel oral anticoagulant (NOAC).^7^ Appropriate control of anticoagulants is important, especially in patients with chronic kidney disease. In the present case, the risk factors were considered to be an older age, chronic heart disease, chronic kidney disease, and high blood pressure. There was no recurrence after switching to edoxaban, an NOAC.

The renal function usually improves quickly in AKI once the cause is removed. If renal dysfunction persists for a long time, it often leads to chronic kidney disease. The RIFLE classification of AKI is “end‐stage renal failure” once renal replacement therapy is required for 3 months or longer.^8^ Among most patients with WRN, the serum creatinine either stabilizes or improves within a few weeks after over‐anticoagulation.^7^ However, some patients may experience little to no recovery of the kidney function. Indeed, in the study cited above, of the nine patients with biopsy‐proven WRN, five patients did not regain their previous kidney function.^2^

In the present case, WRN was confirmed by a renal biopsy, and the patient had been clearly oliguric and needed hemodialysis until 3 months after the onset. However, the renal function was markedly improved by 4 months after the onset, and he was able to be weaned from hemodialysis. As in this case, there was a previous report wherein hemodialysis was continued due to WRN but was withdrawn 6‐10 weeks later because the renal function had improved.^9^, ^10^ In contrast to other AKIs, WRN may delay the renal function improvement. While the exact reason for this is unclear, the time needed for the physical obstruction of the renal tubules to improve may be involved.

Similarly, rhabdomyolysis is known to associate with tubular obstruction and cause acute kidney injury. In this disease, the exact mechanism has not been elucidated, myoglobin forming a complex with the Tamm‐Horsfall protein obstructs renal tubules and causes renal ischemia and oxidative stress.^11^ In warfarin‐related nephropathy, red blood cells obstruct renal tubules. There is possibility that this difference affects the time it takes to recover from kidney damage.

In a previous report, WRN resulted in end‐stage renal failure and continued dialysis; however, there has been no report, which describes that the renal function was observed for a long time after the introduction of dialysis. Since this is a relatively new disease concept, there is no consensus regarding the prognosis of the renal function, and more cases will need to be accumulated in future. Renal dysfunction due to WRN may improve over time, and long‐term follow‐up is important after the initiation of dialysis.

## CONFLICT OF INTEREST

The authors declare that they have no competing interests.

## AUTHOR CONTRIBUTIONS

EN, TT, and KM: collected the clinical data, administered the therapy, and interpreted the clinical course. EN: wrote the manuscript. MF, SR, and MM: supervised the therapy and wrote the manuscript. All the authors have contributed to the preparation of the manuscript. All the authors read and approved the final manuscript.

## CONSENT TO PUBLISH

The patient provided informed consent for publication to our hospital in written form.

## Data Availability

The data regarding the case belongs to clinical and laboratory charts stored in the hospital repository and cannot be shared.
